# Dynamin II is required for 17β-estradiol signaling and autophagy-based ERα degradation

**DOI:** 10.1038/srep23727

**Published:** 2016-03-24

**Authors:** Pierangela Totta, Claudia Busonero, Stefano Leone, Maria Marino, Filippo Acconcia

**Affiliations:** 1 Department of Sciences, Section Biomedical Sciences and Technology, University Roma Tre, Viale Guglielmo Marconi, 446, I-00146, Rome, Italy

## Abstract

17β-estradiol (E2) regulates diverse physiological effects, including cell proliferation, by binding to estrogen receptor α (ERα). ERα is both a transcription factor that drives E2-sensitive gene expression and an extra-nuclear localized receptor that triggers the activation of diverse kinase cascades. While E2 triggers cell proliferation, it also induces ERα degradation in a typical hormone-dependent feedback loop. Although ERα breakdown proceeds through the 26S proteasome, a role for lysosomes and for some endocytic proteins in controlling ERα degradation has been reported. Here, we studied the role of the endocytic protein dynamin II in E2-dependent ERα signaling and degradation. The results indicate that dynamin II siRNA-mediated knock-down partially prevents E2-induced ERα degradation through the inhibition of an autophagy-based pathway and impairs E2-induced cell proliferation signaling. Altogether, these data demonstrate that dynamin II is required for the E2:ERα signaling of physiological functions and uncovers a role for autophagy in the control of ERα turnover.

The physiological effects of the sex steroid hormone 17β-estradiol (E2) are mediated by the estrogen receptors (*i.e.,* ERα and ERβ). In target cells, E2-activated ERs work by stimulating estrogen-response-element (ERE) and non-ERE containing gene transcription in the nucleus, as well as by mediating the induction of diverse extra-nuclear cytoplasmic signaling cascades (*e.g.,* PI3K/AKT pathway)^1^. It is now accepted that these two apparently separable molecular mechanisms integrate in a single pathway that transduces the E2-included stimulus into the regulation of diverse cellular processes (*e.g.,* cell migration, proliferation and apoptosis), both in cell lines and in animal models^2^,^3^.

Indeed, by binding ERα, E2 elicits cell proliferation through up-regulation of the cell cycle regulating gene cyclin D1 and the anti-apoptotic gene B-cell lymphoma 2 (Bcl-2), which either contribute to DNA synthesis and cell cycle progression^4^,^5^. The E2-induced increase in cyclin D1 and Bcl-2 transcription is controlled by the PI3K/AKT pathway^5^,^6^. This extra-nuclear cascade is rapidly activated because E2 engages the membrane localized ERα, which in turn docks to the insulin-like growth factor receptor 1 (IGF-1R) and triggers AKT phosphorylation^7^,^8^. E2-activated AKT controls ERα phosphorylation at serine residue 118 (S118)^9^. S118 phosphorylated ERα then enhances E2:ERα-mediated ERE-containing gene expression^10^, which further contributes to E2-dependent proliferative stimuli. In parallel to these molecular events, E2 also triggers ERα degradation, which primarily occurs through the 26S proteasome^11^ and is required to synchronize the cell response to environmental E2 fluctuations^12^,^13^.

Recently, we have reported that, in addition to the 26S proteasome, lysosomal function controls E2-induced ERα degradation and cell proliferation^14^. Moreover, by performing a siRNA-based screening, we implicated diverse endocytic proteins (*e.g.,* clathrin, caveolin-1 and caveolin-2) in the regulation of both E2-induced cell proliferation and ERα degradation in breast cancer cells^15^,^16^. Interestingly, we also found that dynamin II (DynII) may affect E2-induced receptor breakdown^16^.

DynII is a GTPase involved in the regulation of diverse physiological functions in cells. Indeed, DynII is required for endocytic processes because it allows the pinching off of nascent vesicles from intracellular membranes^17^. Moreover, DynII activity and function is crucial for cell migration, cell proliferation and programmed cell death^17^,^18^,^19^. Thus, although E2- and DynII-regulated cellular processes overlap^1^,^2^,^3^,^17^,^18^,^19^, whether DynII and ERα intersect in the regulation of E2 signaling to cell functions is completely unknown at present.

Here, we investigated the possible role of DynII in the E2-dependent regulation of cell proliferation and report that DynII expression is necessary for the E2:ERα signaling that drives cells to proliferate. Moreover, we unexpectedly identify a DynII-dependent role for autophagy in ERα degradation.

## Results

### Dynamin II depletion perturbs E2-induced cell proliferation signaling

**Figure 1:**
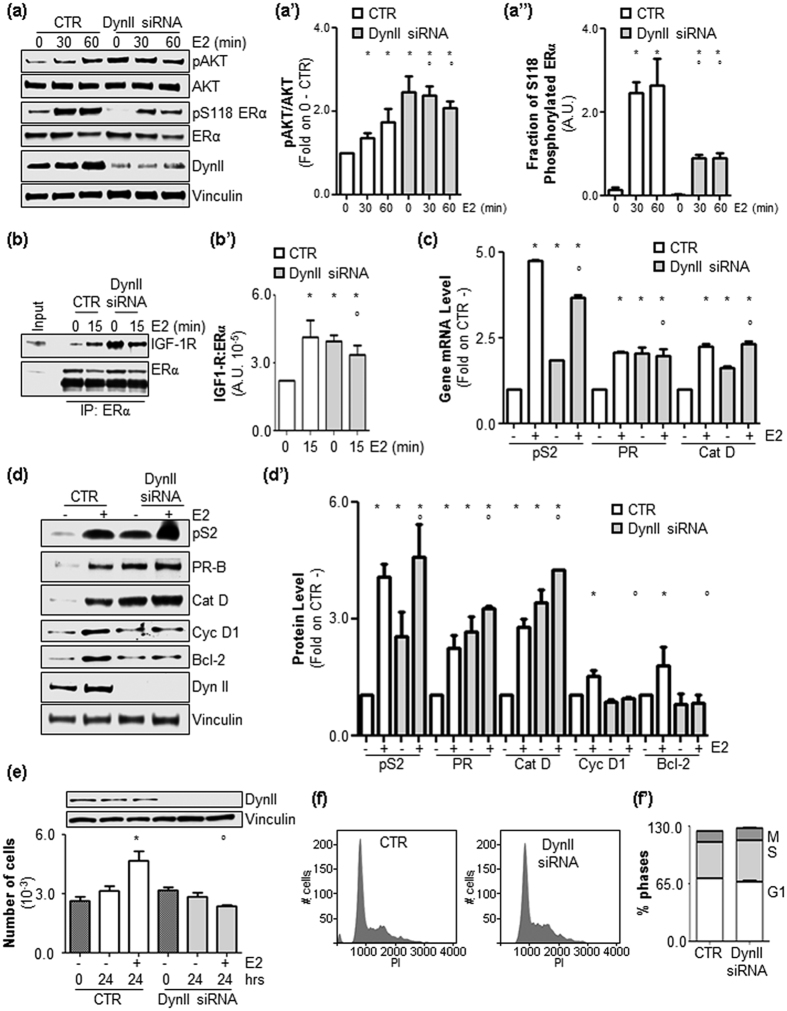
The role of DynII in ERα nuclear and extra-nuclear activities and in E2-induced cell proliferation. (**a**) Western blotting and relative densitometric analyses (**a’, a”**) of AKT and ERα S118 phosphorylation in MCF-7 control (CTR) and DynII knock-down cells treated with E2 (10 nM) at different time points. (**b**) ERα:IGF-1R co-immunoprecipitation and relative densitometric analyses (**b’**) in MCF-7 control (CTR) and DynII knock-down cells treated with E2 (10 nM) at the indicated time points. The loading control was done by evaluating vinculin expression in the same filter. *indicates significant differences with respect to the control (0) sample; °indicates significant differences with respect to the corresponding E2 sample. (**c**) RT-qPCR analysis of pS2/TIFF (pS2), progesterone receptor (PR) and cathepsin D (Cat D) mRNA expression normalized to the GAPDH mRNA expression in MCF-7 control (CTR) and DynII knock-down cells treated with E2 (10 nM) for 24 hrs. *indicates significant differences with respect to the control (CTR−); °indicates significant differences with respect to the E2 CTR sample. Western blotting (**d**) and relative densitometric (d’) analyses of pS2/TIFF (pS2), progesterone receptor (PR), cathepsin D (Cat D), cyclin D1 (Cyc D1) and Bcl-2 expression levels in MCF-7 control (CTR) and DynII knock-down cells treated with E2 (10 nM–24 hrs). The loading control was done by evaluating tubulin or vinculin expression in the same filter. *indicates significant differences with respect to the control (CTR−) sample; °indicates significant differences with respect to the corresponding E2 CTR sample. (**e**) The number of MCF-7 control (CTR) and DynII knock-down cells treated with E2 (10 nM–24 hrs). *indicates significant differences with respect to the control (−); °indicates significant differences with respect to the E2 sample; Time 0 corresponds to plated cells. (**f**) Representative distribution of two experiments performed in MCF-7 control (CTR) and DynII knock-down cells in the different phases of the cell cycle and relative quantitation (**f’**).

Initial experiments were performed to evaluate the impact of DynII knock-down (KD) on E2-induced PI3K/AKT pathway activation in ductal carcinoma cells (MCF-7 cells). Figure 1a,a’” show that depletion of DynII strongly reduced the ability of E2 to increase both AKT and ERα S118 phosphorylation. Because AKT phosphorylation was basally increased in DynII KD cells (Fig. 1a,a’”) and AKT activation depends on the ability of E2 to rapidly associate with the insulin-like growth factor receptor 1 (IGF-1R)^7^, we next evaluated ERα:IGF-1R interaction under DynII depleted conditions (Supplementary Fig. 1A). Figure 1b,b’ show that the E2-triggered increase in ERα:IGF-1R complex formation was prevented in DynII KD MCF-7 cells. Notably, the basal ERα:IGF-1R interaction was strongly increased under basal conditions when the DynII cellular levels were reduced (Fig. 1b,b’). ERα S118 phosphorylation is required for full receptor transcriptional activity^10^. Thus, experiments were next performed to evaluate if DynII KD could affect ERα-dependent gene transcription. Q-PCR and Western blotting analyses performed on control (CTR) and MCF-7 DynII KD cells revealed that reduction in DynII cellular levels deregulates the ability of E2 to induce the expression of ERE-containing genes (*e.g.,* presenelin2 [pS2], progesterone receptor [PR] and cathepsin D [Cat D]) (Fig. 1c,d,d’). Interestingly, DynII KD not only reduced E2-dependent pS2, PR and Cat D gene transcription but also altered their basal intracellular levels (Fig. 1c,d,d’). In addition to ERE-containing genes, the E2:ERα complex activates non-ERE promoters^1^. Also the depletion of DynII from MCF-7 cells prevented the ability of E2 to induce the up-regulation of cyclin D1 (Cyc D1) and Bcl-2 protein levels (Fig. 1d,d’). Cyc D1 and Bcl-2 contribute to E2-induced cell cycle progression^4^,^5^. Thus, experiments were next performed to evaluate the impact of DynII depletion on E2-induced proliferation. Figure 1e shows that E2 lost its ability to increase cell number in MCF-7 DynII KD cells. Remarkably, DynII KD did not significantly change the basal cell cycle distribution in MCF-7 cells (Fig. 1f,f’).

Overall these results show that in MCF-7 cells, DynII depletion increases the basal AKT phosphorylation state, abolishes the ability of E2 to increase ERα S118 phosphorylation and AKT activation, alters the basal and E2-induced mechanism of ERα:IGF-1R interaction, deregulates ERα transcriptional activity and prevents E2 proliferative stimuli.

### Dynamin II depletion and autophagy inhibition induces ERα stabilization

**Figure 2:**
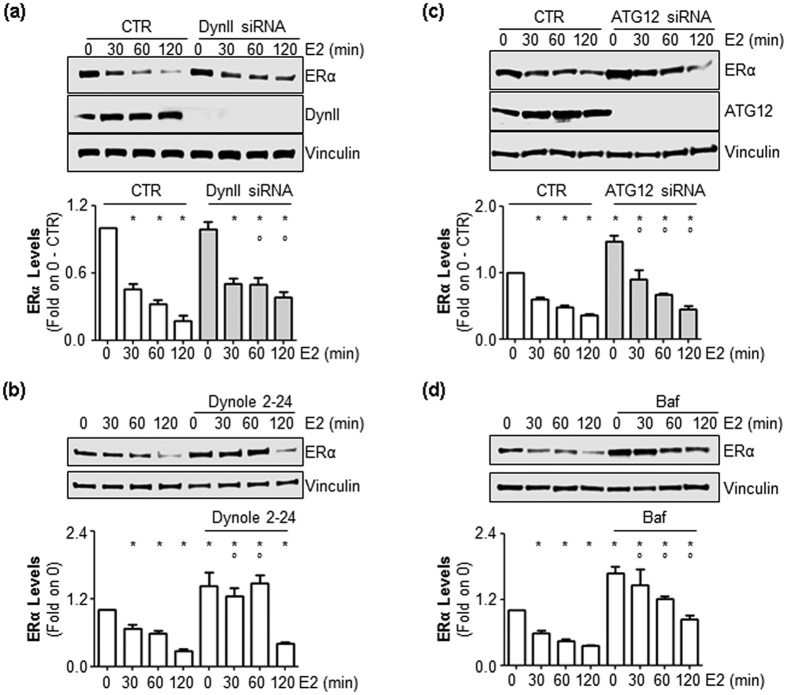
The role of DynII and autophagic flux on E2-induced ERα degradation. (**a**) Western blotting analysis and relative densitometric analyses of ERα cellular levels in MCF-7 control (CTR), DynII (**a**) or ATG12 (**c**) knock-down cells and in dynole 2–24 (5 μM) (**b**) or in bafilomycin A1 (Baf) (100 nM) (**d**) pre-treated cells evaluated in the presence of E2 (10 nM) at different time points. The loading control was done by evaluating vinculin expression in the same filter. *indicates significant differences with respect to the control (0) sample; °indicates significant differences with respect to the corresponding E2 sample.

In parallel to the induction of cell proliferation, E2 triggers ERα degradation in the typical feedback loop that occurs for other mitogen growth factors as well^11^,^20^. Moreover, the E2-activated PI3K/AKT pathway protects the receptor from proteolytic breakdown^16^,^21^. Because DynII KD prevents E2-induced AKT phosphorylation (Fig. 1), we next evaluated the impact of DynII depletion on E2-induced ERα degradation in MCF-7 cells. Surprisingly, we found that reduction in DynII cellular levels in MCF-7 cells partially dampened the effect of E2 in reducing ERα cellular content observed in CTR cells (Fig. 2a). Moreover, pre-treatment of MCF-7 cells with the DynII inhibitors dynole 2–24^22^ or dynasore^23^ also reduced E2-induced ERα degradation (Fig. 2b and supplementary Fig. 1B, respectively).

The 26S proteasome, lysosomes and autophagolysosomes control protein degradation in cells^24^,^25^. Because ERα stability is controlled by the 26S proteasome^11^ and lysosomes^14^ and because a cross-talk between ERα and autophagy has recently been identified^26^, we hypothesized a role for an autophagic pathway in ERα degradation. Thus, we genetically or chemically inhibited the autophagic flux in MCF-7 cells. Depletion of ATG12, a critical component of the autophagic machinery^27^, as well as the pre-treatment of MCF-7 cells with bafilomycin A1 (Baf), an inhibitor of the vacuolar (V)-ATPase^28^, partially prevented the E2-induced reduction in ERα intracellular levels (Fig. 2c,d, respectively). Similar results have been obtained also in another ERα-positive breast cancer cell line (*i.e.,* T47D-1, supplementary Fig. 1C). Notably, interference with both DynII and autophagy increased basal ERα content in MCF-7 cells (Fig. 2). Thus, DynII and an autophagic pathway are, at least in part, necessary for ERα control of intracellular levels in breast cancer cells.

### Autophagy controls basal ERα degradation

**Figure 3:**
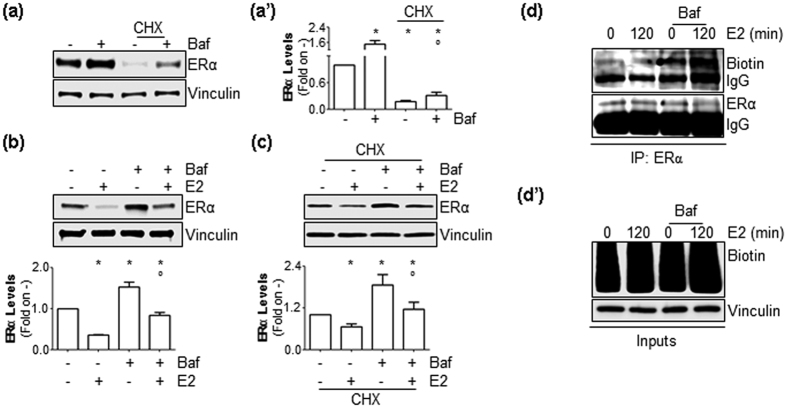
The role of autophagic flux on neo-synthesized and mature ERα. (**a**) Western blotting analysis and relative densitometric analyses (**a’**) of ERα cellular levels in MCF-7 cells treated with bafilomycin A1 (Baf) (100 nM–2 hrs) evaluated in the presence or absence of cycloheximide (CHX) (1 μg/ml–6 hrs). (**b**,**c**) Western blotting analysis and relative densitometric analyses of mature ERα content in MCF-7 cells kept in methionine-free medium for 24 hrs and treated for 2 hrs with E2 (10 nM) in the presence or in the absence of bafilomycin A1 (Baf) (100 nM–2 hrs) with or without cycloheximide (CHX) (1 μg/ml–6 hrs) administration. The loading control was done by evaluating vinculin expression in the same filter. *indicates significant differences with respect to the control (−) sample; °indicates significant differences with respect to the corresponding E2 sample. (**d**) Immunoprecipitation analysis of neo-synthesized ERα cellular levels in MCF-7 cells treated for 2 hrs with E2 (10 nM) in the presence or absence of bafilomycin A1 (Baf) (100 nM–2 hrs). (**d’**) Western blotting analysis of biotin-labelled cellular proteins. The loading control was done by evaluating vinculin expression in the same filter.

ERα is a rapidly turned over protein, and its breakdown occurs both on the pre-formed and the neo-synthesized (*i.e.,* native) receptor^29^. Thus, experiments were next performed to understand on which receptor pool the observed effects of autophagy inhibition could occur. To study the pre-formed receptor, MCF-7 cells were treated for 24 hrs with the protein synthesis inhibitor cycloheximide (CHX), which reduced ERα intracellular levels (Fig. 3a). Interestingly, treatment of MCF-7 cells with Baf both in the presence and absence of CHX increased the basal receptor intracellular content (Fig. 3a). Two hours of E2 treatment both in the presence and absence of CHX administration induced a significant reduction in total ERα cellular content (Fig. 3b,c). Remarkably, this reduction was equally prevented when MCF-7 cells were also treated with Baf (Fig. 3b,c). On the other hand, to study the neo-synthesized receptor^29^, we took advantage of a non-radioactive assay (Click-it®) that labels native proteins with biotin (Fig. 3d’ and supplementary Fig. 2)^30^. As previously reported^21^,^29^, 2 hours of E2 treatment reduced the levels of neo-synthesized ERα (Fig. 3d). Interestingly, Baf treatment increased the levels of the native receptor irrespective of E2 administration (Fig. 3d). Therefore, ERα degradation (*i.e.,* turnover) proceeds at least in part through an autophagy-based pathway that mainly controls basal ERα breakdown.

### E2 inhibits the autophagic flux

**Figure 4:**
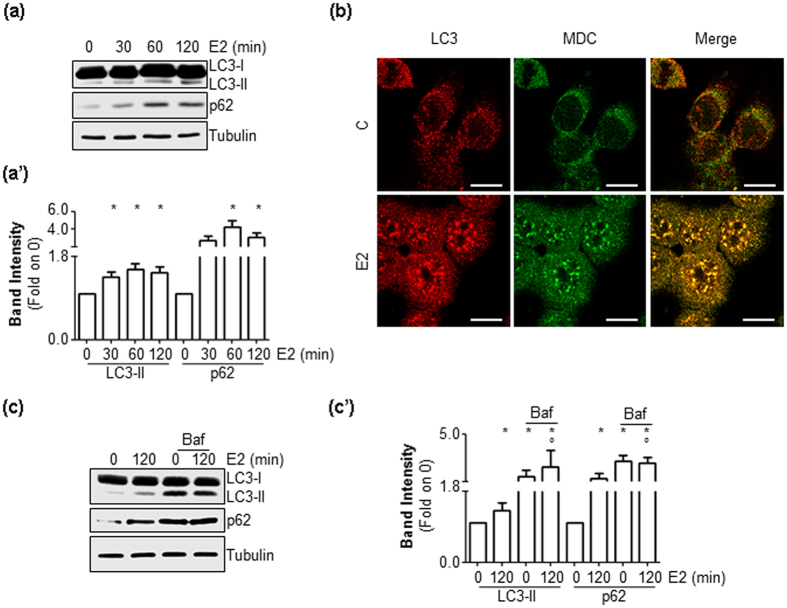
The effect of E2 on autophagosome number. Western blotting analysis and relative densitometric analyses of LC3 and p62 cellular levels in MCF-7 cells treated with E2 at different time points (**a,a’**) or treated for 120 min with E2 in the presence or in the absence of bafilomycin A1 (Baf) (100 nM–2 hrs) (**c,c’**). For LC3 quantitation, the formula LC3-II/(LC3-I + LC3-II) has been applied. The loading control was done by evaluating vinculin expression in the same filter. *indicates significant differences with respect to the control (−) sample; °indicates significant differences with respect to the corresponding E2 sample. (**b**) LC3 (red signal) and monodansylcadaverine (MDC) (green signal) immunofluorescence staining in MCF-7 cells treated with E2 (10 nM) for 120 min. The scale bar represents 10 microns.

Because an autophagy-based pathway is involved in the regulation of ERα intracellular levels (Figs 2 and 3), we next evaluated whether E2 could affect its activation state. When autophagosomes form, LC3-I is converted to LC3-II by lipidation. LC3-II remains in the autophagosomes until it is degraded into autophagolysosomes. Consequently, we evaluated the cellular amount of LC3-II [*i.e.,* LC3-II/(LC3-I + LC3-II)] as a marker of autophagosome number^25^. Figure 4a,a’ show that E2 induced a rapid increase in LC3-II cellular content. Moreover, treatment of MCF-7 cells with E2 (2 hrs) led to an increase in LC3 staining, which was superimposable onto the signal derived from monodansylcadaverine (MDC, Fig. 4b), an autofluorescent molecule that stains autophagic vacuoles^25^.

p62, also known as sequestrosome, is an autophagy receptor that addresses soluble proteins (*i.e.,* cargo molecule) to autophagolysosomes for degradation. p62 associates with LC3-II and undergoes degradation in autophagolysosomes, together with the transported cargo. Thus, p62 can be considered a substrate of autophagy, which is accumulated when autophagy is impaired^25^. Thus, we evaluated the effect of E2 on p62 intracellular levels in MCF-7 total cellular lysates extracted in the presence of 1% SDS^25^. Figure 4a,a’ show that E2 induced a time-dependent increase in p62 cellular content.

To test if LC3-II and p62 increase represented an induction of the autophagic process in E2-treated cells or, on the contrary, an inhibition of LC3-II and p62 degradation by the autophagolysosome, MCF-7 cells were treated with E2 in the presence of Baf^25^. As shown in Fig. 4c,c’ Baf administration in MCF-7 cells resulted in no further significant increase in LC3 and p62 levels. Altogether, these data indicate that E2 increases autophagosome number and inhibits the later stages of the autophagic flux.

### ERα localizes to autophagosomes

**Figure 5:**
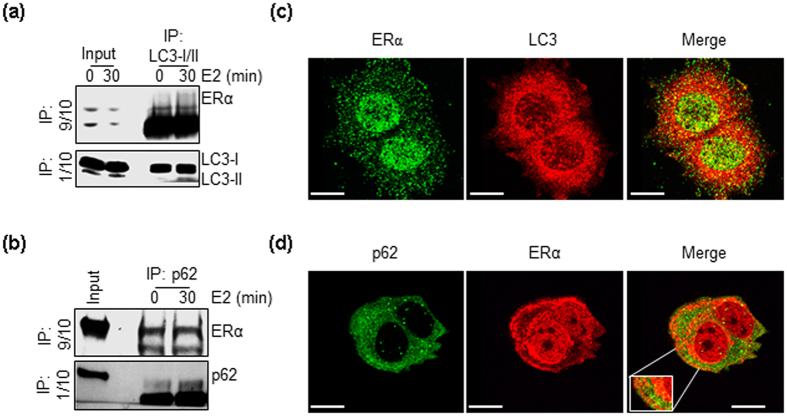
ERα association with autophagosomal markers. Immunoprecipitation analysis of ERα:LC3 (**a**) and ERα:p62 (**b**) association in MCF-7 cells treated with E2 (10 nM) for 30 min. 9/10 of the immunoprecipitation samples was used for evaluating the presence of ERα by Western blotting while 1/10 of the same sample was run on a different gel for normalization. LC3 and ERα (**c**) or p62 and ERα (**d**) immunofluorescence staining in untreated MCF-7 cells. In (**c**), ERα signal was obtained by using anti-ERα F-10 antibody (Santa Cruz Biotechnology), while in (**d**), ERα signal was obtained by using anti-ERα SP-1 antibody (Thermo Fisher). The scale bar represents 10 microns.

Next, we evaluated whether ERα localizes to autophagosomes in MCF-7 cells. Figure 5a,b show that ERα co-precipitated with LC3 or p62 in un-stimulated MCF-7 cells subjected to immunoprecipitation with an anti-LC3 or an anti-p62 antibody, respectively. Moreover, E2 administration did not change the amount of ERα immunoprecipitated with LC3 or p62 (Fig. 5a,b). In line with these biochemical results, confocal microscopy experiments performed with anti-ERα antibodies (*i.e.*, *C*-terminal domain-directed antibodies) that highlight the extra-nuclear located ERα in a different manner^31^,^32^ revealed that LC3 and ERα (Fig. 5c), as well as p62 and ERα (Fig. 5d) staining signals merge in un-treated MCF-7 cells. Thus, ERα localizes to autophagosomes in an E2-independent manner.

### Dynamin II KD prevents autophagy in an E2-independent manner

**Figure 6:**
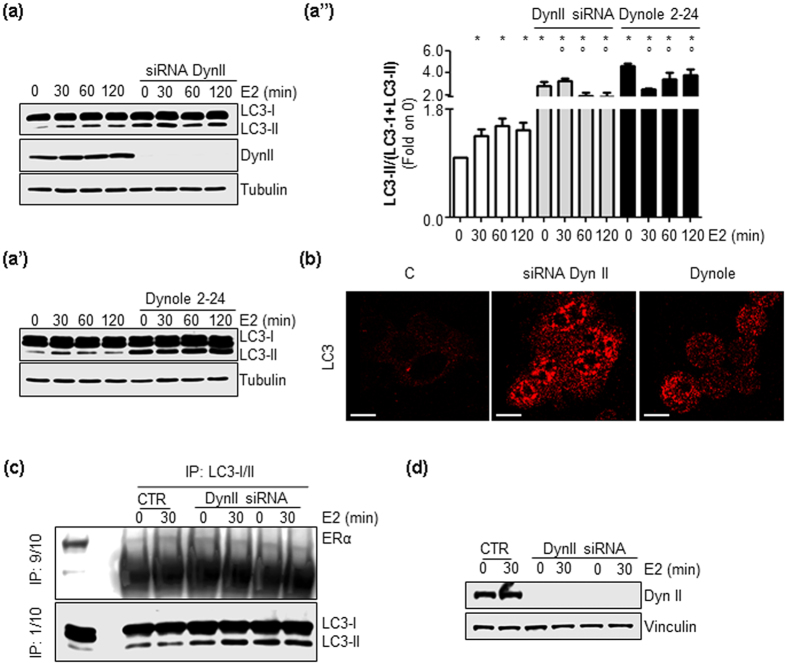
The role of DynII on autophagy. Western blotting analysis and relative densitometric analyses of LC3 cellular levels in MCF-7 cells treated with E2 at different time points both in the presence or absence of siRNA against dynamin II (DynII siRNA) (**a,a”**) or dynole 2–24 treatment (5 μM) (**a’,a”**). LC3 quantitation was performed using the formula LC3-II/(LC3-I+LC3-II). The loading control was done by evaluating tubulin expression in the same filter. * indicates significant differences with respect to the control (−) sample; ° indicates significant differences with respect to the corresponding E2 sample. (**b**) LC3 immunofluorescence staining in untreated MCF-7 cells both in the presence or in the absence of siRNA against dynamin II (DynII siRNA) or dynole 2–24 treatment (5 μM). The scale bar represents 10 microns. Immunoprecipitation analysis of ERα:LC3 association (**c**) in MCF-7 control (CTR) and DynII knock-down cells (**d**) treated with E2 (10 nM) for 30 min. 9/10 of the immunoprecipitation samples was used for evaluating the presence of have ERα by Western blotting, while 1/10 of the same sample was run on a different gel for normalization.

Because a role for DynII in inhibiting autophagy has been reported^33^,^34^ and our observation that DynII and autophagic flux inhibition both partially prevent E2-induced ERα degradation (Figs 2 and 3), we finally determined whether DynII depletion blocked autophagy in MCF-7 cells. Figure 6a,a” show that DynII KD increased the cellular amount of LC3-II in un-treated MCF-7 cells. Remarkably, while E2 induced a rapid and persistent accumulation of LC3-II in MCF-7 CTR cells, the hormone did not increase it further under DynII depleted conditions (Fig. 6a,a”). Moreover, similar results were obtained when MCF-7 cells were pre-treated with the DynII inhibitors dynole 2–24^22^ and dynasore^23^ (Fig. 6a’ and supplementary Fig. 1D, respectively). Finally, we determined the ERα:LC3 association under DynII depleted conditions (Fig. 6c,d). As reported in Fig. 6c, the depletion of DynII KD both in the presence and absence of E2 did not increase the amount of ERα immunoprecipitated by LC3 antibody. Overall, these data indicate that DynII depletion increases the number of autophagosomes, irrespective of E2 and does not affect the ERα:LC3 association.

## Discussion

In this study, we report that DynII is required for activation of E2-evoked cell proliferation signaling and for the control of autophagy-mediated ERα degradation. Therefore, our findings demonstrate that DynII plays a dual role in the regulation of ERα-based pathways in breast cancer cells (*e.g.,* signaling and degradation) and further support the concept that endocytic proteins regulate E2:ERα-mediated physiological functions.

In cells, the molecular mechanisms elicited by E2 that are required for the induction of cell proliferation are mediated by ERα. Although this hormone receptor was long considered a nuclear protein that regulates gene expression, it is now clear that ERα also works as a plasma membrane-localized receptor^2^,^3^,^5^,^21^. As for other hormones that bind to membrane receptors (*e.g.,* receptor tyrosine kinases - RTKs)^17^, E2 induces ERα degradation while it activates intracellular signaling circuitries required for hormone-regulated physiological effects. As a consequence, different investigators have suggested an endocytic trafficking of E2-activated membrane-localized ERα, as it occurs for activated RTKs^16^,^35^,^36^,^37^.

Support for this notion came from recent observations that, besides 26S proteasome-mediated degradation, ERα breakdown also occurs in lysosomes^14^. Because lysosomes are endocytic stations^17^, we hypothesized a role for endocytic proteins in the control of receptor degradation. Thus, we applied a library of siRNA oligonucleotides designed against endocytic proteins to breast cancer cells (*i.e.,* MCF-7 cells) and studied E2-induced ERα degradation^16^. By silencing the expression of clathrin heavy chain (CHC), adaptor protein 2 (AP2), caveolin-1 and caveolin-2, we reported that E2 loses its ability to induce the PI3K/AKT pathway. This event leads to reduced ERα S118 phosphorylation that subsequently leads to a reduction in receptor-mediated ERE-containing gene transcription and a reduction in E2-dependent cyclin D1 and Bcl-2 up-regulation. In parallel, the lack of PI3K/AKT pathway-dependent ERα S118 phosphorylation renders the receptor more susceptible to degradation. Consequently, E2-dependent cell proliferation is prevented^15^,^16^.

Here, we report that the DynII depletion-dependent phenotype in MCF-7 cells is superimposable to the one detected when either CHC, AP-2, caveolin-1 or caveolin-2 intracellular levels are reduced. In particular, DynII expression is required for E2-dependent activation of the PI3K/AKT pathway, ERα S118 phosphorylation, ERE and non-ERE gene transcription that all contribute to E2-dependent regulation of cell proliferation. Interestingly, the basal increase in PI3K/AKT cascade activation under DynII KD conditions (*i.e.,* increased AKT activation and IGF-1:ERα association) can be explained by considering that, since inhibition of DynII blocks most endocytic events, the activity of a plasma membrane endocytic-dependent signaling pathway is augmented in cells when DynII is silenced^17^. Thus, a unique plasma membrane pathway includes the functions of all these endocytic proteins and impinges on the control of E2-induced cell proliferation.

Aside from the effect of the depletion of the above mentioned endocytic proteins^15^,^16^, DynII KD also reduces the ability of E2 to trigger ERα degradation. Because DynII depletion has been shown to inhibit autophagy^33^,^34^, we reasoned that the DynII-dependent control of ERα content could occur through autophagic degradation. Here, we demonstrate that E2-induced ERα degradation is partially prevented when cellular levels of ATG12, a critical component of the autophagy machinery, are reduced, as well as when autophagy is blocked by bafilomycin A1 (Baf)^25^. Accordingly, the same effect was observed in MCF-7 cells when DynII was genetically or chemically inhibited, thus confirming that DynII inhibition prevents autophagic flux^33^,^34^. Interestingly, autophagy inhibition also results in an increased basal amount of cellular ERα. Steady-state cellular ERα content is the result of degradative pathways (*e.g.,* 26S proteasome) that insist on the pools of both neo-synthesized and mature ERα fractions^11^. We report that Baf administration to cells reduces the degradation of both ERα pools under basal conditions. On the contrary, only E2-induced degradation of the neo-synthesized receptor is prevented when autophagy is inhibited. In line with this finding, ERα localizes to the autophagosomes. During the completion of the autophagic flux, soluble proteins (*i.e.,* autophagic cargoes) can bind to p62, which in turn docks at LC3-II and shuttles them to autophagolysosomes for subsequent degradation in a non-canonical process named selective autophagy^38^. Because we observed that ERα co-precipitates with both LC3 and p62 in MCF-7 cells, it is possible that ERα autophagosomal localization depends on the ability of the receptor to form a complex with both p62 and LC3. Thus, we conclude that a DynII-dependent selective autophagy-based pathway is involved in the control of ERα turnover.

Another interesting aspect of this work is the effect of E2 on autophagy. It has been reported that E2 can modulate autophagy, with some evidence suggesting that E2 activates autophagy^39^,^40^, whereas other investigators report that E2 blocks the autophagic flux^41^,^42^. The autophagic flux begins with the formation of a double membrane (*i.e.,* phagophore) that engulfs organelles, cytoplasmic portions and/or specific proteins (*i.e.,* autophagic cargoes) and originates a vesicle called an autophagosome. The subsequent fusion of autophagosomes with lysosomes forms the autophagolysosomes where autophagic cargoes are hydrolyzed^27^. Here we show that E2 increases the autophagosome number in MCF-7 cells and that this number is not additionally increased by the administration of Baf, as occurs when a molecule activates autophagy^25^. Therefore, E2-induced autophagosome biogenesis does not depend on an increase in the rate of phagophore formation but rather occurs because E2 inhibits the completion of the autophagic flux at its terminal stage. Moreover, E2 does not change the amount of ERα that co-precipitates with LC3 or p62. Thus, we conclude that E2 does not address ERα to autophagosomes and blocks autophagy.

The presented results additionally disclose a paradoxical circumstance for which E2 inhibits autophagy and induces cell proliferation, while DynII depletion impairs both autophagy and E2-induced cell proliferation. Nonetheless, such contradiction can be reconciled by envisioning a situation in which, by blocking autophagy, E2 triggers cell survival because it impedes autophagic degradation of neo-synthesized ERα, which in turn is available to sustain E2-induced signaling to cell proliferation. On the contrary, the inhibition of autophagy, as determined by DynII silencing or by other stimuli^43^, would block breast cancer cell proliferation, possibly through an increase in both the pre-formed and the neo-synthesized ERα pools. In this regard, our findings unveil a more general concept concerning the relations between the control of ERα intracellular levels and E2-induced cell proliferation in breast cancer cells. Indeed, the depletion of endocytic proteins that either stabilize (*e.g.,* DynII) or de-stabilize (*e.g.,* CHC)^16^ ERα causes the same blocking effect in E2-induced cell proliferation. Similarly, the loss of ERα in receptor-positive cell lines as well as ERα re-expression in ER-negative cells results in artificial cell lines that fail to proliferate in response to E2^44^,^45^. Furthermore, certain ligands bound to ERα (*e.g.,* 4OH-tamoxifen and ICI 182,280) prevent E2-induced cell proliferation but either reduce (4OH-tamoxifen) or accelerate (ICI 182,280) ERα turnover^11^.

In turn, it seems that whatever causes imbalance in ERα levels (*e.g.,* ligands/molecules or pathways) has the potential to inhibit E2-dependent proliferation of breast cancer cells. Thus, the physiological control of intracellular ERα levels possesses an intrinsic weakness, which breast cancer cells control to fuel proliferation. Consequently, this point of weakness might be exploited for therapeutic purposes aimed at blocking ERα-positive breast cancer progression.

## Methods

### Cell culture and reagents

17β-estradiol, DMEM (with and without phenol red) and fetal calf serum were purchased from Sigma-Aldrich (St. Louis, MO). The Bradford protein assay kit, as well as anti-mouse and anti-rabbit secondary antibodies, were obtained from Bio-Rad (Hercules, CA). Antibodies against ERα (HC-20 rabbit; F-10 mouse), cyclin D1 (H-295 rabbit), Bcl-2 (C2 mouse), progesterone receptor (C20 rabbit), cathepsin D (H75 rabbit), pS2 (FL-84 rabbit), dynamin II (C-18 goat), p62/SQSTM (D-3 mouse) and anti-goat secondary antibody (sc-2020) were obtained from Santa Cruz Biotechnology (Santa Cruz, CA) and anti-vinculin, anti-tubulin and anti-LC3 antibodies were purchased from Sigma-Aldrich (St. Louis, MO). Anti-phospho-AKT, anti-AKT, anti-phospho-S118 ERα, and anti-IGF-1R were purchased from Cell Signaling Technology, Inc. (Beverly, MA). Anti-biotin-HRP was purchased from Thermo Fisher Scientific (Walthman, MA, USA). Chemiluminescence reagent for Western blotting was obtained from BioRad Laboratories (Hercules, CA, USA). Dynole 2–24 was purchased from Abcam (USA). All other products were from Sigma-Aldrich. Analytical- or reagent-grade products, without further purification, were used. The identities of all of the cell lines that were used (*i.e.,* human breast carcinoma cells [MCF-7; T47D-1]) were verified by STR analysis (BMR Genomics, Italy).

### RNA interference experiments, cellular and biochemical assays, RNA isolation, qPCR and cell cycle analysis

The silencing of DynII or ATG12 in MCF-7 cells was conducted by transient transfection of Dharmacon smart pool siRNA oligos (final concentration 4 nM in 2 mL of a six well plate). For all siRNA experiments control cells (CTR) have been transfected with the same amount of transfection medium (RNAi Max+Optimem) but the siRNA oligonucleotide, as described elsewhere^15^,^16^,^20^,^46^. All other assays were performed as previously described^15^,^16^.

### Confocal microscopy analysis

Assays were performed as previously described^16^. Anti-LC3, anti-p62/SQSTM and anti-ERα were diluted 1:200 and incubated for 2 hours at R.T. MDC staining was performed by administrating this molecule to cells (50 μM) for 1 hour before fixation.

### Metabolic Labeling of Newly Synthesized Proteins with L-azidohomoalanine AHA

MCF-7 cells were maintained for 24 hrs in methionine-free medium. E2 and inhibitors were added together with L-azidohomoalanine (AHA, Supplementary Fig. 2). After 2 hrs, cells were lysed in YY buffer (50 mM HEPES at pH 7.5, 10% glycerol, 150 mM NaCl, 1% Triton X-100, 1 mM EDTA, 1 mM EGTA). Two hundred μg were used for the click-reaction with biotin-alkyne and then used to immunoprecipitate ERα. Biotin-labelled proteins were detected by Western blotting with anti-biotin-HRP antibody. Each step in the labeling procedure was performed according to the manufacturer instructions of the Click-iT® metabolic labeling reagents for proteins kit (Invitrogen) (Supplementary Fig. 2).

### Statistical analysis

A statistical analysis was performed using the ANOVA (One-way analysis of variance and Tukey’s as post-test) test with the InStat version 3 software system (GraphPad Software Inc., San Diego, CA). Densitometric analyses were performed using the freeware software ImageJ, by quantifying the band intensity of the protein of interest with respect to the relative loading control band (*i.e.,* vinculin or tubulin) intensity. In all analyses, *p* values < 0.01 were considered significant, except for densitometric analyses where *p* values < 0.05 were considered significant.

## Additional Information

**How to cite this article**: Totta, P. *et al.* Dynamin II is required for 17β-estradiol signaling and autophagy-based ERα degradation. *Sci. Rep.* **6**, 23727; doi: 10.1038/srep23727 (2016).

## Supplementary Material

Supplementary Information

## References

[b1] Ascenzi P., Bocedi A. & Marino M. Structure-function relationship of estrogen receptor alpha and beta: impact on human health. Mol Aspects Med 27, 299–402 (2006). 16914190 10.1016/j.mam.2006.07.001

[b2] Pedram A., Razandi M., Lewis M., Hammes S. & Levin E. R. Membrane-localized estrogen receptor alpha is required for normal organ development and function. Developmental Cell, doi: 10.1016/j.devcel.2014.04.016 (2014). PMC4062189 24871949

[b3] Adlanmerini M. *et al.* Mutation of the palmitoylation site of estrogen receptor alpha *in vivo* reveals tissue-specific roles for membrane versus nuclear actions. Proc Natl Acad Sci USA 111, E283–290 (2014). 24371309 10.1073/pnas.1322057111 PMC3896153

[b4] Castoria G. *et al.* PI3-kinase in concert with Src promotes the S-phase entry of oestradiol-stimulated MCF-7 cells. EMBO J 20, 6050–6059 (2001). 11689445 10.1093/emboj/20.21.6050 PMC125704

[b5] Acconcia F. *et al.* Survival versus apoptotic 17beta-estradiol effect: role of ER alpha and ER beta activated non-genomic signaling. J Cell Physiol 203, 193–201 (2005). 15389627 10.1002/jcp.20219

[b6] Pedram A., Razandi M., Aitkenhead M., Hughes C. C. & Levin E. R. Integration of the non-genomic and genomic actions of estrogen. Membrane-initiated signaling by steroid to transcription and cell biology. J Biol Chem 277, 50768–50775 (2002). 12372818 10.1074/jbc.M210106200

[b7] Song R. X. *et al.* The role of Shc and insulin-like qrowth factor 1 receptor in mediating the translocation of estrogen receptor a to the plasma membrane. Proceedings of the National Academy of Sciences of the United States of America 101, 2076–2081 (2004). 14764897 10.1073/pnas.0308334100 PMC357054

[b8] Simoncini T. *et al.* Interaction of oestrogen receptor with the regulatory subunit of phosphatidylinositol-3-OH kinase. Nature 407, 538–541 (2000). 11029009 10.1038/35035131 PMC2670482

[b9] Acconcia F. *et al.* An inherent role of integrin-linked kinase-estrogen receptor alpha interaction in cell migration. Cancer Res 66, 11030–11038 (2006). 17108142 10.1158/0008-5472.CAN-06-2676

[b10] Ali S., Metzger D., Bornert J. M. & Chambon P. Modulation of transcriptional activation by ligand-dependent phosphorylation of the human oestrogen receptor A/B region. EMBO J 12, 1153–1160 (1993). 8458328 10.1002/j.1460-2075.1993.tb05756.x PMC413317

[b11] Leclercq G., Lacroix M., Laios I. & Laurent G. Estrogen receptor alpha: impact of ligands on intracellular shuttling and turnover rate in breast cancer cells. Curr Cancer Drug Targets 6, 39–64 (2006). 16475975 10.2174/156800906775471716

[b12] Metivier R. *et al.* Estrogen receptor-alpha directs ordered, cyclical, and combinatorial recruitment of cofactors on a natural target promoter. Cell 115, 751–763 (2003). 14675539 10.1016/s0092-8674(03)00934-6

[b13] Reid G. *et al.* Cyclic, proteasome-mediated turnover of unliganded and liganded ERalpha on responsive promoters is an integral feature of estrogen signaling. Mol Cell 11, 695–707 (2003). 12667452 10.1016/s1097-2765(03)00090-x

[b14] Totta P., Pesiri V., Marino M. & Acconcia F. Lysosomal Function Is Involved in 17beta-Estradiol-Induced Estrogen Receptor alpha Degradation and Cell Proliferation. PLoS One 9, e94880 (2014). 24736371 10.1371/journal.pone.0094880 PMC3988130

[b15] Totta P., Gionfra F., Busonero C. & Acconcia F. Modulation of 17beta-Estradiol Signaling on Cellular Proliferation by Caveolin-2. J Cell Physiol, doi: 10.1002/jcp.25218 (2015). 26480297

[b16] Totta P., Pesiri V., Enari M., Marino M. & Acconcia F. Clathrin Heavy Chain Interacts With Estrogen Receptor alpha and Modulates 17beta-Estradiol Signaling. Mol Endocrinol 29, 739–755 (2015). 25860340 10.1210/me.2014-1385 PMC5414742

[b17] Sigismund S. *et al.* Endocytosis and signaling: cell logistics shape the eukaryotic cell plan. Physiol Rev 92, 273–366 (2012). 22298658 10.1152/physrev.00005.2011 PMC5614474

[b18] Soulet F., Schmid S. L. & Damke H. Domain requirements for an endocytosis-independent, isoform-specific function of dynamin-2. Exp Cell Res 312, 3539–3545 (2006). 16938290 10.1016/j.yexcr.2006.07.018

[b19] Fish K. N., Schmid S. L. & Damke H. Evidence that dynamin-2 functions as a signal-transducing GTPase. J Cell Biol 150, 145–154 (2000). 10893263 10.1083/jcb.150.1.145 PMC2185575

[b20] Sigismund S. *et al.* Clathrin-mediated internalization is essential for sustained EGFR signaling but dispensable for degradation. Developmental Cell 15, 209–219 (2008). 18694561 10.1016/j.devcel.2008.06.012

[b21] La Rosa P., Pesiri V., Leclercq G., Marino M. & Acconcia F. Palmitoylation Regulates 17beta-Estradiol-Induced Estrogen Receptor-alpha Degradation and Transcriptional Activity. Mol Endocrinol 26, 762–774 (2012). 22446104 10.1210/me.2011-1208 PMC5417099

[b22] Robertson M. J., Deane F. M., Robinson P. J. & McCluskey A. Synthesis of Dynole 34-2, Dynole 2–24 and Dyngo 4a for investigating dynamin GTPase. Nature protocols 9, 851–870 (2014). 24651498 10.1038/nprot.2014.046

[b23] Macia E. *et al.* Dynasore, a cell-permeable inhibitor of dynamin. Dev Cell 10, 839–850 (2006). 16740485 10.1016/j.devcel.2006.04.002

[b24] Powers E. T. & Balch W. E. Diversity in the origins of proteostasis networks–a driver for protein function in evolution. Nat Rev Mol Cell Biol 14, 237–248 (2013). 23463216 10.1038/nrm3542 PMC3718298

[b25] Klionsky D. J. *et al.* Guidelines for the use and interpretation of assays for monitoring autophagy. Autophagy 8, 445–544 (2012). 22966490 10.4161/auto.19496 PMC3404883

[b26] Felzen V. *et al.* Estrogen receptor alpha regulates non-canonical autophagy that provides stress resistance to neuroblastoma and breast cancer cells and involves BAG3 function. Cell Death Dis 6, e1812, doi: 10.1038/cddis.2015.181 (2015). 26158518 PMC4650728

[b27] Maiuri M. C., Zalckvar E., Kimchi A. & Kroemer G. Self-eating and self-killing: crosstalk between autophagy and apoptosis. Nat Rev Mol Cell Biol 8, 741–752 (2007). 17717517 10.1038/nrm2239

[b28] Yoshimori T., Yamamoto A., Moriyama Y., Futai M. & Tashiro Y. Bafilomycin A1, a specific inhibitor of vacuolar-type H(+)-ATPase, inhibits acidification and protein degradation in lysosomes of cultured cells. J Biol Chem 266, 17707–17712 (1991). 1832676

[b29] Laios I. *et al.* Role of the proteasome in the regulation of estrogen receptor alpha turnover and function in MCF-7 breast carcinoma cells. J Steroid Biochem Mol Biol 94, 347–359 (2005). 15857754 10.1016/j.jsbmb.2005.02.005

[b30] Dieterich D. C., Link A. J., Graumann J., Tirrell D. A. & Schuman E. M. Selective identification of newly synthesized proteins in mammalian cells using bioorthogonal noncanonical amino acid tagging (BONCAT). Proc Natl Acad Sci USA 103, 9482–9487 (2006). 16769897 10.1073/pnas.0601637103 PMC1480433

[b31] Welsh A. W. *et al.* Cytoplasmic Estrogen Receptor in Breast Cancer. Clin Cancer Res 18, 118–126 (2012). 21980134 10.1158/1078-0432.CCR-11-1236 PMC3263348

[b32] Dan P., Cheung J. C., Scriven D. R. & Moore E. D. Epitope-dependent localization of estrogen receptor-alpha, but not -beta, in en face arterial endothelium. Am J Physiol Heart Circ Physiol 284, H1295–1306 (2003). 12531733 10.1152/ajpheart.00781.2002

[b33] Durieux A. C. *et al.* A centronuclear myopathy–dynamin 2 mutation impairs autophagy in mice. Traffic 13, 869–879 (2012). 22369075 10.1111/j.1600-0854.2012.01348.x

[b34] Schulze R. J. *et al.* Lipid droplet breakdown requires dynamin 2 for vesiculation of autolysosomal tubules in hepatocytes. J Cell Biol 203, 315–326 (2013). 24145164 10.1083/jcb.201306140 PMC3812963

[b35] Kisler K., Chow R. H. & Dominguez R. Fluorescently-Labeled Estradiol Internalization and Membrane Trafficking in Live N-38 Neuronal Cells Visualized with Total Internal Reflection Fluorescence Microscopy. J Steroids Horm Sci Suppl 12, doi: 10.4172/2157-7536.S12-002 (2013). PMC3863688 24353903

[b36] Hammes A. *et al.* Role of endocytosis in cellular uptake of sex steroids. Cell 122, 751–762 (2005). 16143106 10.1016/j.cell.2005.06.032

[b37] Pietras R. J. & Szego C. M. Specific internalization of estrogen and binding to nuclear matrix in isolated uterine cells. Biochem Biophys Res Commun 123, 84–91 (1984). 6477590 10.1016/0006-291x(84)90383-8

[b38] Rogov V., Dotsch V., Johansen T. & Kirkin V. Interactions between autophagy receptors and ubiquitin-like proteins form the molecular basis for selective autophagy. Mol Cell 53, 167–178 (2014). 24462201 10.1016/j.molcel.2013.12.014

[b39] Zielniok K., Motyl T. & Gajewska M. Functional interactions between 17 beta -estradiol and progesterone regulate autophagy during acini formation by bovine mammary epithelial cells in 3D cultures. BioMed research international 2014, 382653, doi: 10.1155/2014/382653 (2014). PMC4033348 24895572

[b40] Fan D. *et al.* Estrogen receptor alpha induces prosurvival autophagy in papillary thyroid cancer via stimulating reactive oxygen species and extracellular signal regulated kinases. J Clin Endocrinol Metab 100, E561–571 (2015). 25594859 10.1210/jc.2014-3257

[b41] Hsieh D. J. *et al.* 17beta-Estradiol and/or Estrogen Receptor beta Attenuate the Autophagic and Apoptotic Effects Induced by Prolonged Hypoxia Through HIF-1alpha-Mediated BNIP3 and IGFBP-3 Signaling Blockage. Cell Physiol Biochem 36, 274–284 (2015). 25967966 10.1159/000374070

[b42] Cook K. L. *et al.* Knockdown of estrogen receptor-alpha induces autophagy and inhibits antiestrogen-mediated unfolded protein response activation, promoting ROS-induced breast cancer cell death. FASEB J 28, 3891–3905 (2014). 24858277 10.1096/fj.13-247353 PMC4139896

[b43] Maycotte P. & Thorburn A. Targeting autophagy in breast cancer. World journal of clinical oncology 5, 224–240 (2014). 25114840 10.5306/wjco.v5.i3.224 PMC4127596

[b44] Oesterreich S. *et al.* Re-expression of estrogen receptor alpha in estrogen receptor alpha-negative MCF-7 cells restores both estrogen and insulin-like growth factor-mediated signaling and growth. Cancer Res 61, 5771–5777 (2001). 11479214

[b45] Jiang S. Y. & Jordan V. C. Growth regulation of estrogen receptor-negative breast cancer cells transfected with complementary DNAs for estrogen receptor. J Natl Cancer Inst 84, 580–591 (1992). 1556769 10.1093/jnci/84.8.580

[b46] Sigismund S. *et al.* Threshold-controlled ubiquitination of the EGFR directs receptor fate. EMBO J 32, 2140–2157 (2013). 23799367 10.1038/emboj.2013.149 PMC3730230

