# Supplementary material for: Dynamin II is required for 17β-estradiol signaling and autophagy-based ERα degradation
Source: Sci Rep. 2016 Mar 24;6:23727. doi: 10.1038/srep23727 (PMC4806323; doi:10.1038/srep23727)

Dynamin II is required for 17β-estradiol signaling and autophagy-based ERα degradation.

Pierangela Totta[1], Claudia Busonero[1], Stefano Leone[1], Maria Marino[1] and Filippo Acconcia[1*].

[1]Department of Sciences, Section Biomedical Sciences and Technology, University Roma Tre, Viale Guglielmo Marconi, 446, I-00146, Rome, Italy.

Supplementary Figure Legends.

Figure 1.

(A) Dynamin II expression levels in MCF-7 control (CTR) and DynII knock-down cells treated with E2 (10 nM) for 15 min. Loading control was done by evaluating tubulin expression in the same filter. These samples are the same as the ones used in Figure 1B and served as control of efficient DynII knock-down. Western blotting analysis and relative densitometric analyses of ERα (B) and LC3 (D) cellular levels in MCF-7 cells treated with E2 (10 nM) at different time points both in the presence or in the absence of dynasore (6 μM). LC3 quantitation was done using the formula LC3-II/(LC3-I+LC3-II). Loading control was done by evaluating tubulin expression in the same filter. * indicates significant differences with respect to the control (-) sample; ° indicates significant differences with respect to the corresponding E2 sample. (C) Western blotting analysis and relative densitometric analyses of ERα cellular levels in T47D-1 cells pre-treated with bafilomycin A1 (Baf) (100 nM) evaluated in the presence of E2 (10 nM) at different time points. The loading control was done by evaluating vinculin expression in the same filter. * indicates significant differences with respect to the control (0) sample; ° indicates significant differences with respect to the corresponding E2 sample.

Figure 2.

Schematic of the procedure used for evaluating the effect of bafilomycin A1 on neo-synthesized ERα as depicted in Figure 3D and 3D’.

Supplementary Figure 1

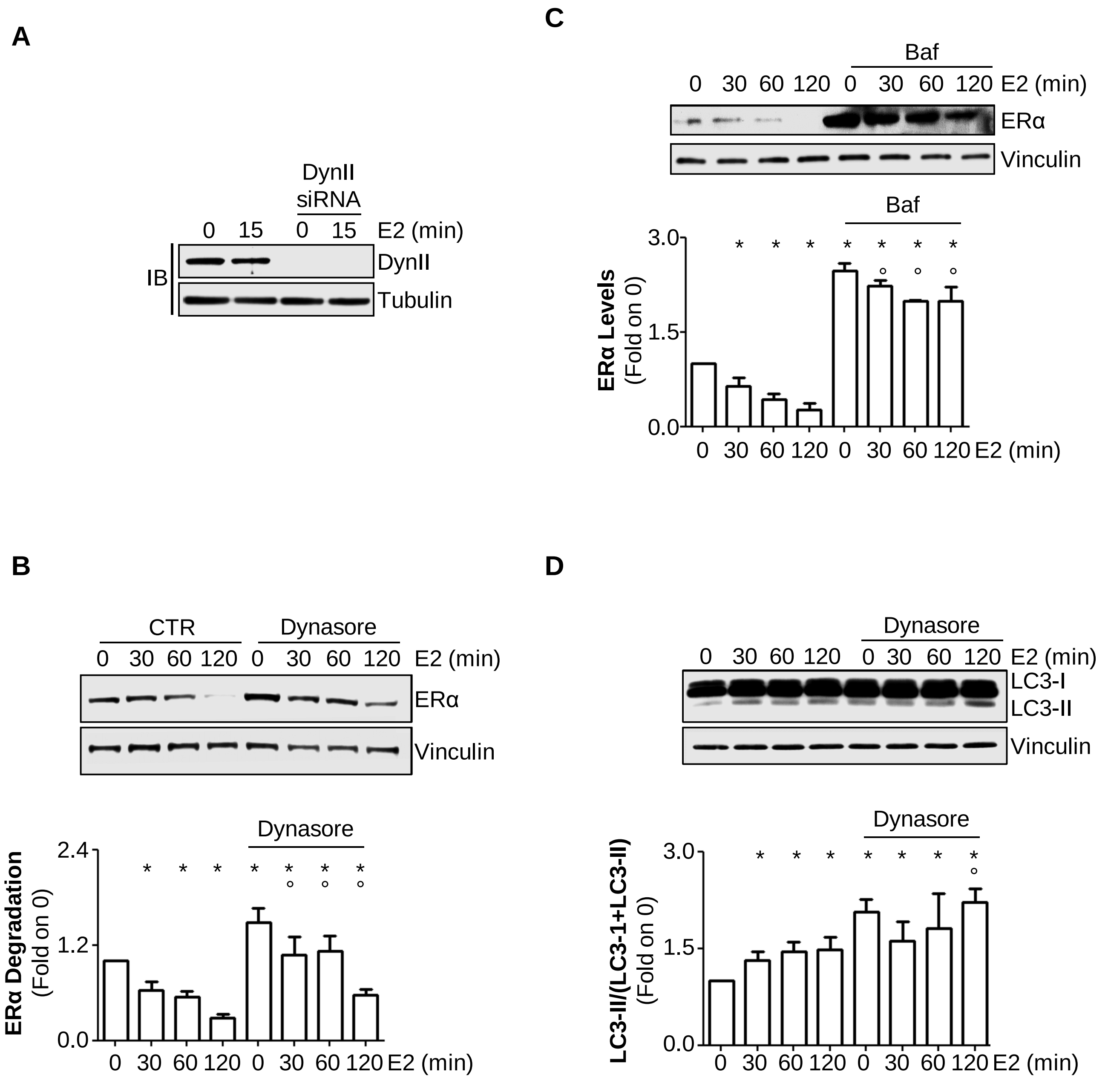

Supplementary Figure 2

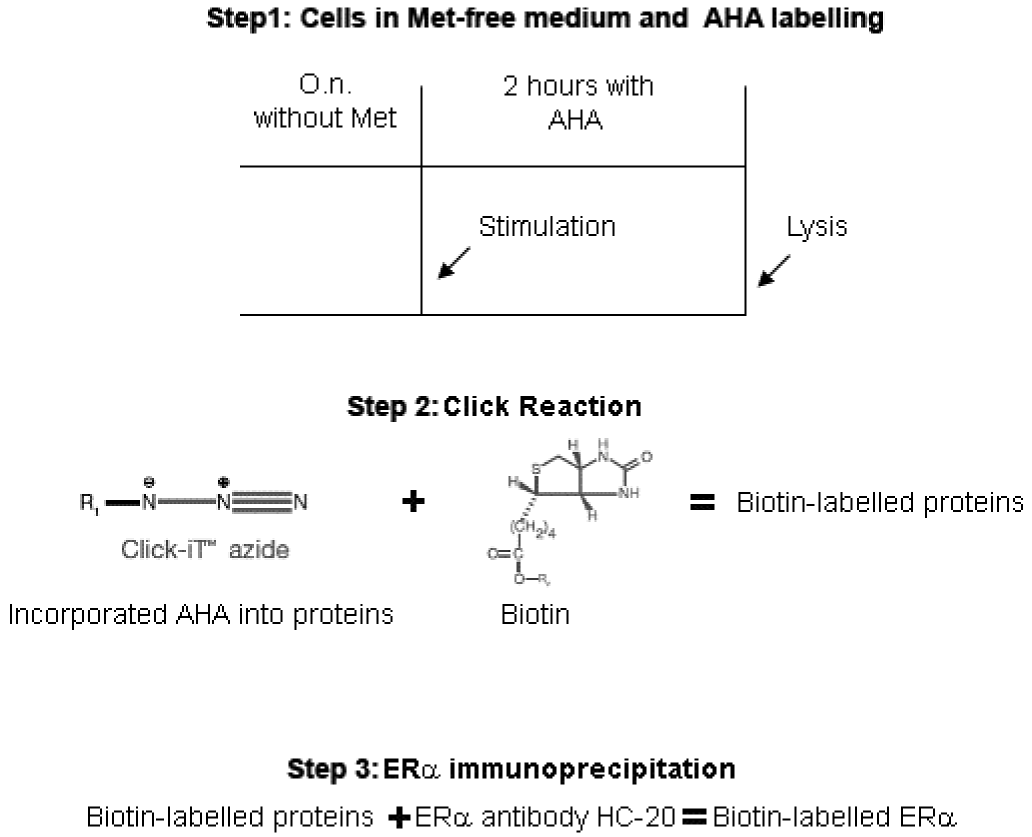

Supplement: Supplementary Information [file srep23727-s1.pdf]
